# DCENet-based low-light image enhancement improved by spiking encoding and convLSTM

**DOI:** 10.3389/fnins.2024.1297671

**Published:** 2024-03-05

**Authors:** Xinghao Wang, Qiang Wang, Lei Zhang, Yi Qu, Fan Yi, Jiayang Yu, Qiuhan Liu, Ruicong Xia, Ziling Xu, Sirong Tong

**Affiliations:** Equipment Management and Unmanned Aerial Vehicle Engineering School, Air Force Engineering University, Xi’an, China

**Keywords:** intensity-to-latency, spiking encoding, low-light enhancement, unpaired image, deep learning

## Abstract

The direct utilization of low-light images hinders downstream visual tasks. Traditional low-light image enhancement (LLIE) methods, such as Retinex-based networks, require image pairs. A spiking-coding methodology called intensity-to-latency has been used to gradually acquire the structural characteristics of an image. convLSTM has been used to connect the features. This study introduces a simplified DCENet to achieve unsupervised LLIE as well as the spiking coding mode of a spiking neural network. It also applies the comprehensive coding features of convLSTM to improve the subjective and objective effects of LLIE. In the ablation experiment for the proposed structure, the convLSTM structure was replaced by a convolutional neural network, and the classical CBAM attention was introduced for comparison. Five objective evaluation metrics were compared with nine LLIE methods that currently exhibit strong comprehensive performance, with PSNR, SSIM, MSE, UQI, and VIFP exceeding the second place at 4.4% (0.8%), 3.9% (17.2%), 0% (15%), 0.1% (0.2%), and 4.3% (0.9%) on the LOL and SCIE datasets. Further experiments of the user study in five non-reference datasets were conducted to subjectively evaluate the effects depicted in the images. These experiments verified the remarkable performance of the proposed method.

## 1 Introduction

The lack of illumination leads to the loss of image information, which severely affects the execution of visual tasks, e.g., face recognition, object detection, dataset preparation, and autonomous driving (Li J. et al., 2021; Liu et al., 2021; Tang et al., 2022; Guo et al., 2023). Capturing images in low-light conditions poses a challenge owing to the limited aperture size, demand for instantaneous processing, and limited memory resources. To mitigate the issues of structuring and the high expense of research and development associated with hardware, refining images in low-light settings through minimalistic software algorithms aligns better with predictable requirements.

In low-light image enhancement (LLIE), the first effective methods were based on histogram equalization, the Retinex model, gamma transform, and fusion. Fusion-based methods achieve better performance in terms of image indicators, such as brightness and color, through exposure-splicing fusion methods. This method is typically synthesized by collecting images under different exposure conditions (Wang et al., 2016). Another method fuses the illumination map of night and day to enhance the image (Rao et al., 2010); however, such processing generally renders a poor visual effect.

The method based on the Retinex model divides the low illumination image into reflection and illumination components or adds a noise component by constructing a suboptimal problem. The estimated reflection component is considered the result of enhancement. Previous attempts to improve Retinex replaced the logarithmic solution with a typical enhanced Lagrange solver to enhance the image with a long image processing time. However, the variational optimization algorithm has a high computational cost. Moreover, it introduces unnecessary pseudo-details in the image.

The adaptive GAMMA transform can improve an image’s contrast; however, most algorithms of this class still cause local overexposure or underexposure in the enhanced result. As most images are captured in non-uniform lighting conditions, Chen et al. (2022) proposed a naturalness- and information-preserving method for processing them. The MEMBHE algorithm (Dar and Mittal, 2020) improved the functionality of the transform through histogram equalization after multiple exposure smoothing. Nevertheless, it overconsumes memory and requires arduous incremental updates.

Several methods for achieving LLIE with deep learning (DL) have been researched. Among them, supervised learning, a mature and informative DL method typically constructed by an end-to-end network, was the first to be applied to an LLIE field. Low-light net (LLNet) (Lore et al., 2017) was the first end-to-end LLIE network established by constructing a deep auto-encoder structure. MBLLEN (Lv et al., 2018) uses three subnetworks to extract rich image features of different levels and introduces a regional loss function into the network loss function to employ different loss weights for high- and low-light regions. In the same vein, Li et al. (2021) determined that enhancing the low-frequency layer of a low-light image with noise was easier than directly enhancing the whole image. Progressive recursive networks (Cai et al., 2018) were used to perform staging, which is a more efficient method for preserving image details and removing noise. In that method, each subnetwork could better achieve its own function, which was eventually enhanced by gradually improving the quality of the image.

Ke et al. (2020) established an SCIE multiexposure dataset (Ke et al., 2020) consisting of low-contrast images with different exposure levels and their corresponding high-quality reference images. Furthermore, they introduced the high- and low-frequency components of images as prediction targets. A double-exposure fusion algorithm (Ying et al., 2017) was proposed to design the weight matrix of image fusion using an illuminance-estimation technique. Then, a camera response model was introduced to synthesize the multiexposure images. Low- and high-exposure images can also be used to estimate the perceptual gain, signal strength, signal structure, and mean intensity. Perceptual gain suits an underexposed image. The feature fusion and recalibration module (FFRM) (Singh et al., 2024) was proposed to recalibrate and merge the features to provide an enhanced output image. Intrinsic image decomposition (Zhang and Ma, 2023) can be applied to the fusion of multiexposure to generate HDR images.

Retinex was combined with DL for enhanced performance (Chen et al., 2018; Zhang Y. et al., 2019; Tang et al., 2023). The attention mechanism was combined with the Retinex model to construct DL networks for enhancement (Chen et al., 2022). A decomposition network (Liu et al., 2023) was developed with a self-supervised fine-tuning strategy that achieved promising performance without manual hyperparameter tuning. Different sensitivities relate to different regions. The low-rank regularized Retinex model (Bao et al., 2022) can represent the image as low-rank decomposition, preserve the image details and high-frequency information, and improve the visual quality of the image. A plug-and-play framework for image enhancement and noise removal based on the Retinex theory (Wu et al., 2023) was introduced. Inspired by guided filtering and using synthetic data for network training, Li et al. (2018) designed a lightweight network architecture based on the Retinex theory. By including the unsettling V channel image component in the HSV color space, the component was converted to a reflection component using a DL network (Jiang Z. et al., 2021). Owing to their significant worth, their Retinex and DL-based methods were applied in image dehazing and underwater image enhancement (Xu et al., 2022; Shen et al., 2023).

The development of LLIE in DL is not limited. Creative thinking models, such as those based on unsupervised learning, represented by the unsupervised learning method (Zhu et al., 2020; Li et al., 2021), generative network architecture (Jiang Z. et al., 2021), and normalizing flow (Wang et al., 2022), show the immense research potential of LLIE. The strategy network learns the local exposure sequentially using reinforcement learning for a segmented subimage (Rong et al., 2018). In the generated adversarial network architecture, global–local discriminators (Jiang Z. et al., 2021) were used to ensure that the enhanced results resemble real normal light images. With the strong capability of image generation, diffusion models were applied to LLIE. For example, the pyramid diffusion model (Zhou et al., 2023) was constructed to solve the RGB shift. Moreover, the inference speed of the diffusion model was accelerated. As a scientific structure for image feature extraction, transformers have become some of the most prevalent network structures in vision processing. The regional distributions have been effectively managed, and the histogram loss has been designed in a stage transformer-guided network (Jiang et al., 2023). Half-wavelet attention block and hierarchical M-Net were utilized to improve computation consumption and reserve context information, aided by the DAU block and discrete wavelet transformation (Fan et al., 2022).

Spiking neural networks (SNNs) are frequently employed in numerous pixel-level classification tasks (Martinez-Seras et al., 2023), such as object detection (Zhang et al., 2023b), image segmentation (Zhang et al., 2023a), and anomaly detection (Yusob et al., 2018). Research centered on SNNs includes methods for neural network learning, data coding, and hardware platforms. The learning approaches for SNNs can be divided into supervised and unsupervised learning, which are represented by spike-timing-dependent plasticity (STDP). Spiking encoding, which involves utilizing discrete pulsed signals to convey information, is a method of signal transmission. Neuroscience computing has access to specialized offline or online application-specific integrated circuit platforms, as well as neuromorphic computing cores that can support various learning rules and neuronal models. Nonetheless, spiking neural network research continues to confront significant barriers. The training of the transformed SNN still relies on the backpropagation algorithm of artificial neural networks (ANNs). As the performance difference between the SNN and the core ANN is small, the former cannot provide significant advantages. Moreover, generative tasks, such as LLIE, image patching, multimodal image generation, and network deployment, present significant challenges. As a new neural network structure, the SNN’s internal algorithm can be implemented in LLIE.

The main contributions of this study are as follows: (1) According to the progressive output results with the specified number concluding the embodiment of the image structure characteristics, the application of the SNN in a spiking encoding method for LLIE has distinct advantages in extracting structural features from images (the intensity-to-latency encoding outputs multiple feature maps with structure and specified steps); and (2) a convLSTM structure that can better absorb the features from multiple feature maps. Based on unlabeled, unsupervised, and unpaired image training via simplified DCENet, the proposed structure is improved by spiking encoding and the convLSTM module. The research introduces spiking encoding, which concludes the image’s backbone information to describe the hierarchical information. The rest of the paper follows this structure: Section 2 describes the proposed enhancement method. Section 3 describes the user study and ablation experiment carried out in the study and compares the performance of the proposed method with the state-of-the-art network structure based on seven objective indicators. Section 4 concludes the study and discusses the potential applications.

## 2 Proposed method

### 2.1 DCENet structure

**Figure 1:**
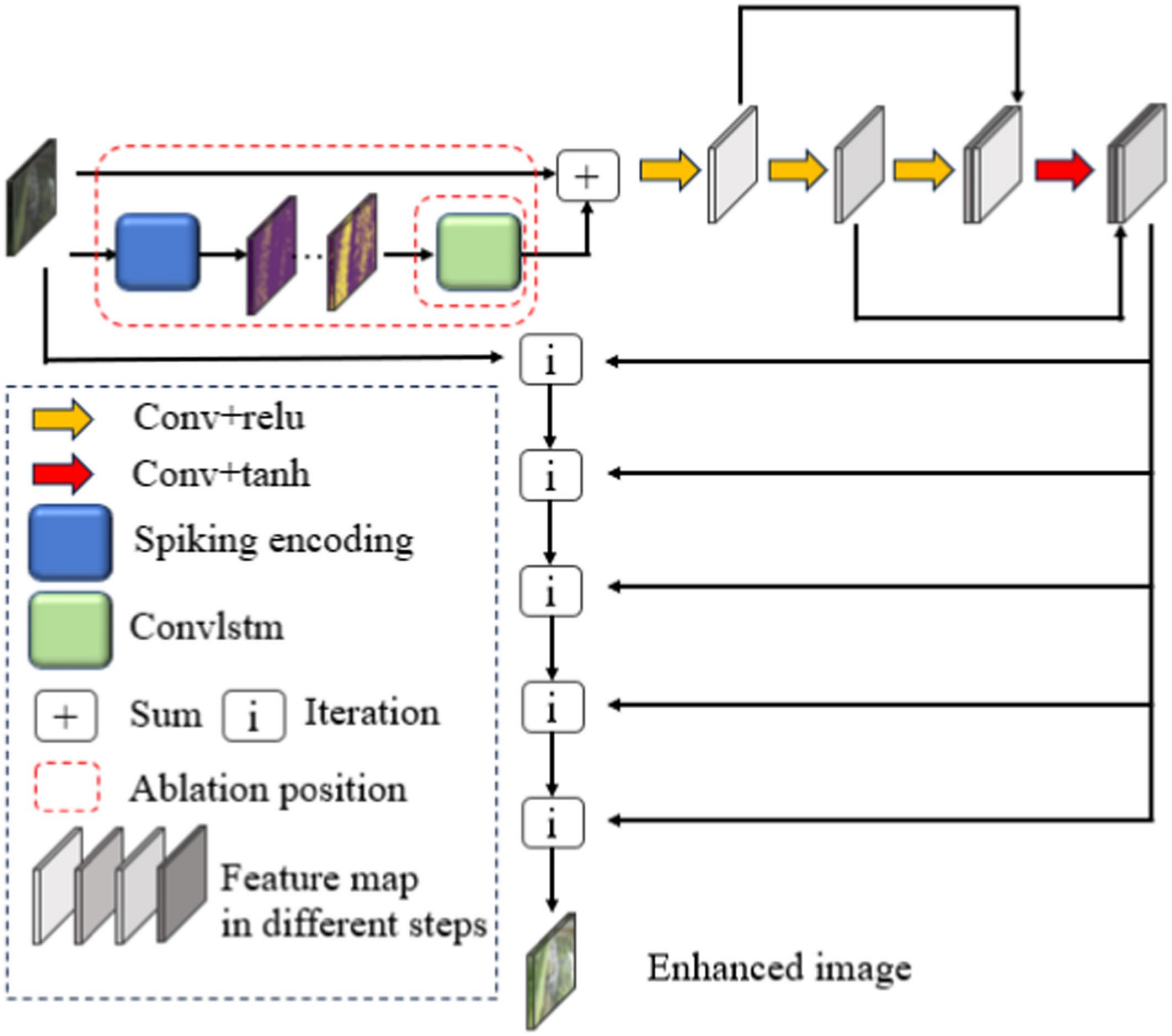
DCENet structure with spiking encoding and convLSTM.

The DCENet structure, as the primary structure used for unsupervised enhancement, divides the LLIE into a high-order iterative process, i.e., the input dark light image is finally enhanced through several iterations of the same operation. Figure 1 depicts the overall enhancement process and part of the ablation study, which can also have a description in literal form. The input passes through the spiking encoding module and ConvLSTM described in subheadings 2.2 and 2.3, respectively, and then through the convolution module containing skip links. The sum module in Figure 1 means a direct overlay between the ConvLSTM’s final output and the input dark light images. The resulting features select the feature graph of a certain channel in order and combine the matrix of the same size in the length and width scale of the output and input images with the initial input tensor according to Equation (1). The matrix is used as the input for the next iteration, and the feature graph of the next channel is selected as needed for the next iteration operation.

Compared with the mathematical relationship represented by the previous gamma transform, the DCENet structure changes the training coefficient of the second term of the right-hand side of Equation (1) into a training coefficient matrix with the same dimensions as those of the input image. This can restrain the problem of over-enhancement or under-enhancement of the image to a certain extent. Finally, the normal brightness area in the image is maintained, and the low illumination area is restored. A is the output of the network, which can be divided into several pieces denoted by $A_{n}$. Based on the number of iterations *n*, the final output enhancement result is $x_{n}$.


(1) $$x_{n}=x_{n-1}+A_{n}\left(x_{n-1}^{2}-x_{n-1}\right)$$

### 2.2 Spiking encoding method

**Figure 2:**
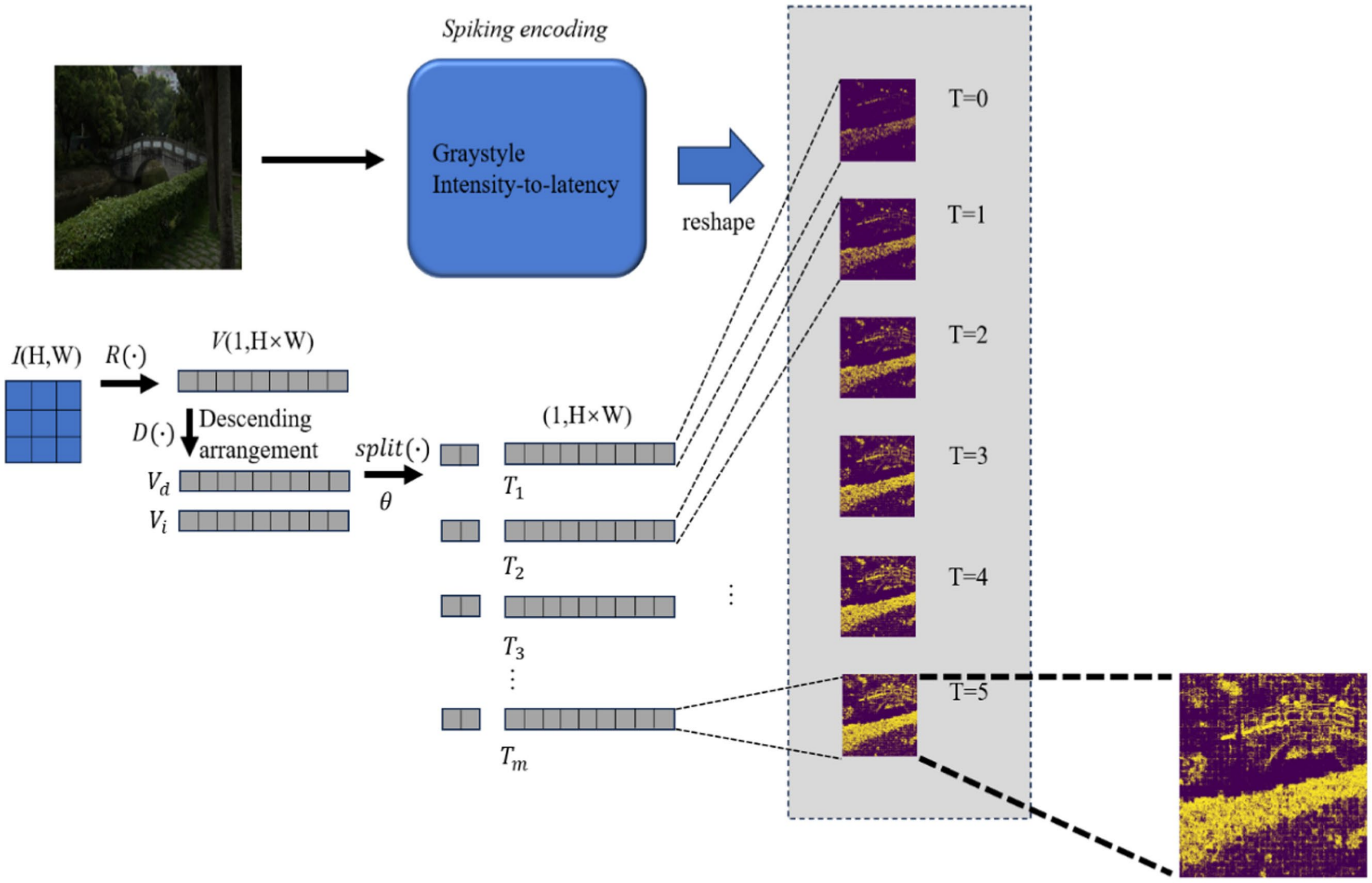
Intensity-to-latency encoding data-flow schematic diagram.

In this study, spiking encoding from the overall DCENet structure equals the intensity-to-latency transform (Mozafari and Ganjtabesh, 2019), as illustrated in Figure 2. First, the intensity-to-latency transform requires an initial parameter, i.e., time step *S*. Then, the grayscale image, which corresponds to a matrix with shape (*H*,*W*), is reshaped to a vector with *H × W* dimensions by *R*(*·*) as illustrated in Equation (2). We named this original vector *V*. For the next step, the vector was arranged in descending order. This procedure generated two vectors with the same dimensions: the first vector is the descending order vector *V_d_*, while the second one is the index vector *V_i_*, corresponding to the index in *V* and this relation is represented by Equation (3).


(2) $$V\left[1,H*W\right]=R\left(I\left[H,W\right]\right)$$

(3) $$V_{d},V_{i}=D\left(V\right)$$

where *K* is the number of non-zero elements in an original vector *V*. The split parameter *θ* is set in Equation (4). *V_d_* and *V_i_* are split into small vector pieces; *θ* decides the shape of these pieces. The small vector piece returns to the dimension *H* × *W*, which is called the spiking encoding vector *T_m_* in Equation (5), with the complementary element filled with 0. The small label *m* ranges from 0 to the time step *S*. The start time step *T_0_* is composed of the value in the first split piece, and the value in *T_0_* is rearranged to the original position in *V* according to *V_i_*. The second time step *T_1_*, which is based on *T_0_*, adds the second small piece, and the value in the second piece is adjusted to the original position in the same way. Thus, the intensity-to-latency transformation is complete. The sequence of outputs *T_m_* is reshaped to similar dimensions as those of the input image, which are denoted by *E_m_*, with the dimensions of (*S*,*H*,*W*). This procedure is formulated as Equation (6). R*(*·*) means the reverse calculation manipulation of *R*(*·*). (Considering the length of the paper, its time step in the figure is set to 6.)


(4) $$\theta =\{\begin{array}{c} \frac{K}{S},\frac{K}{S}>2\\ 2,\frac{K}{S}\leq 2 \end{array}$$

(5) $$T_{m}=\mathrm{split}\left(V_{d},\theta \right)$$

(5) $$E_{m}=R^{*}\left(T_{m},V_{i}\right)$$

### 2.3 ConvLSTM

**Figure 3:**
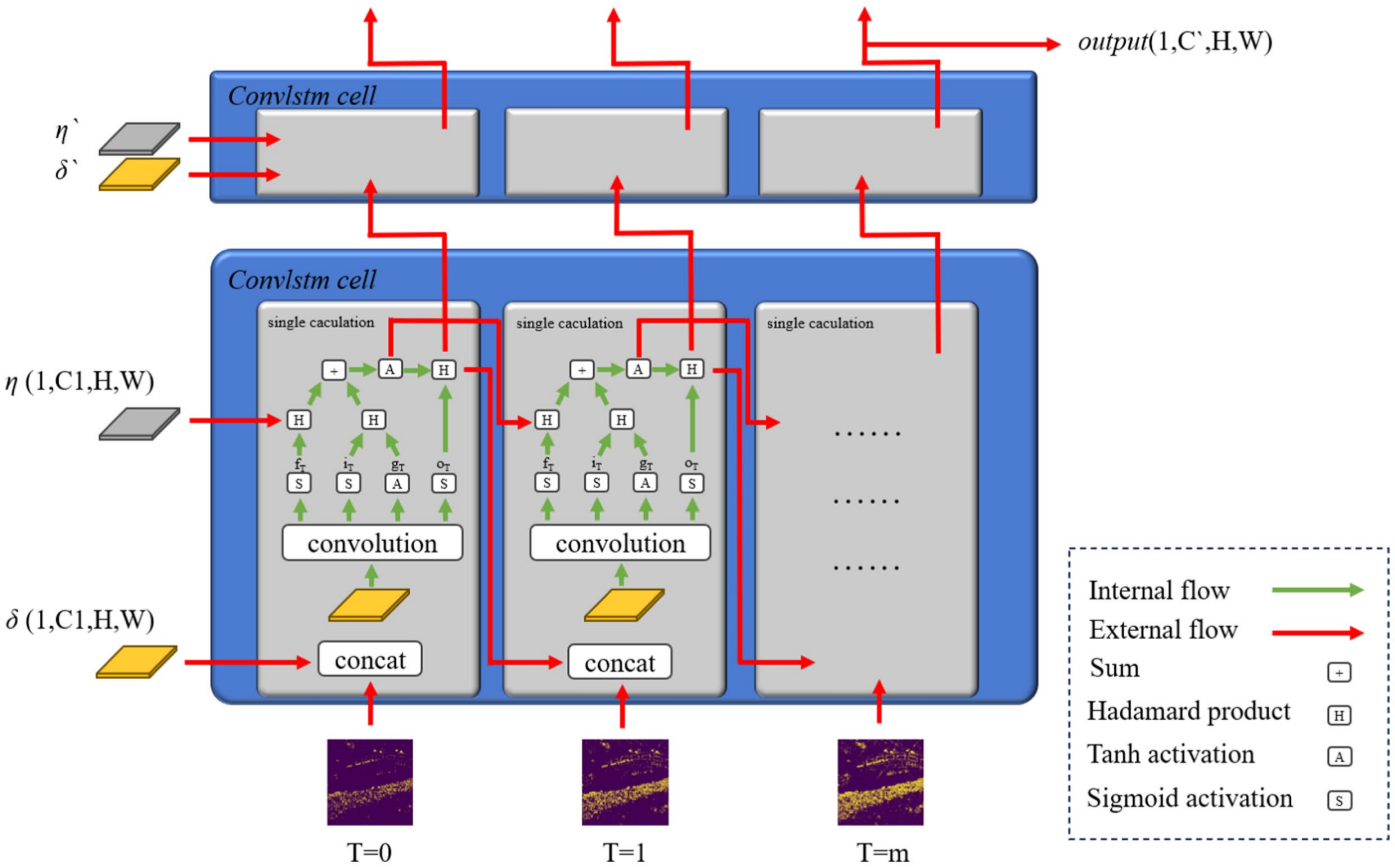
ConvLSTM block data-flow schematic diagram.

The features extracted by the intensity-to-latency transform have certain similarities and differences. These features will constitute an image sequence with fluent features. The convLSTM structure is applicable in this scenario. ConvLSTM is proposed for precipitation nowcasting (Shi et al., 2015), the backbone of which is the recurrent neural network (RNN) for spatiotemporal prediction with convolutional structures. This design is convenient for video and image sequence-related tasks. ConvLSTM is similar to LSTM, which is also called FC-LSTM, and its block structure is illustrated in Figure 3.

The convLSTM computation method is based on LSTM’s gate relationship. The distinction between its different layers is the input and output dimensions. The core of convLSTM is the convLSTM cell, which represents one convLSTM layer. ConvLSTM cell is an RNN-like structure; therefore, a specific hidden-layer parameter called *hidden state* is required. In every convLSTM layer, the hidden state is initialized with a zero element of dimensions (*C*1,*H*,*W*). One of the input sequence time step tensors *I*, which is another input of convLSTM and the outputs from spiking encoding, with dimensions (*C*,*H*,*W*), were concatenated with *δ*. It outputs a combined tensor with dimensions (*C* + *C*1,*H*,*W*), corresponding to the concatenate calculation represented by *concat* in Equation (6), which needs two different variables. The convLSTM cell accepts this combined tensor and outputs the tensor with dimensions (4 × *C*1,*H*,*W*). The outputs were divided into four tensors with dimensions (*C*1,*H*,*W*) for the outputs of different gates: input, forget, and output gates, and a new *δ* for the subsequent layer and input time step. This divided single step is represented by the *split*. The calculation procedure is summarized in Equations 6–10 and Figure 2.


(6) $$mid_{1}=\mathrm{conv}\left(\mathrm{concat}\left(\delta_{t-1},I_{t-1}\right),\dim=1\right)$$

(7) $$c_{i},c_{f},c_{o},c_{g}=\mathrm{split}\left(mid_{1},\mathrm{hidden},\dim=1\right)$$

(8) $$a_{t}=S\left(c_{a}\right),a=i,f,o;g_{t}=A\left(c_{g}\right)$$

(9) $$\eta_{t}=f_{t}{}^{\circ}\eta_{t-1}+i_{t}{}^{\circ}g_{t}$$

(10) $$\delta_{t}=o_{t}{}^{\circ}A\left(\eta_{t}\right)$$

In the convLSTM structure, *δ* and *η*, which are output by one convLSTM cell, pass to the next cell at a certain time. This time corresponds to the next time step in the same layer. This RNN-like network structure will preserve the main features from the previous time step image feature. The *δ* is also output to the convLSTM cell, combined with the new hidden states *δ`* and *η`* in the next layer in the same time step. The overall output of the convLSTM module is the tensor with dimensions (1,*C*`,*H*,*W*), which is labeled *output* in Figure 2. The time step dimension is eliminated with the convLSTM module and *S*(·), sigmoid activation function, and *A*(·), tanh activation function.

### 2.4 The loss items of the DCENet structure

Four loss items, namely spatial consistency loss, color constancy loss, exposure control loss, and illumination smoothness loss, were considered for the convergence of the network. The loss function used by the network is represented by Equation (12). The spatial consistency loss item was calculated by Equation (13). The purpose of setting the spatial consistency loss item was to maintain the difference between the original image and the adjacent area of a pixel in the enhanced image as small as possible. The $\bar{X}$ represents the tensor X after channel averaging and average pooling for every 4 × 4 area. K is the number of pixels after average pooling in one feature map channel. These pixels are separated by a distance of 1, which corresponds to a point assemble called $R\left(i\right)$. This difference logic will enhance the pixel neighborhood within the same spatial structure. By introducing the sum item, the pixel neighborhood consistency can be promoted to the spatial position consistency of the whole image. The setting of this loss item will maintain the spatial consistency of the image before and after enhancement.


(12) $$L=L_{\mathrm{spa}}+L_{\text{exp}}+W_{\mathrm{col}}L_{\mathrm{col}}+W_{tv}L_{tv}$$

(13) $$L_{\mathrm{spa}}=\frac{1}{K}\overset{K}{\underset{i=1}{\sum }}\underset{j\in R\left(i\right)}{\sum }{\left(\left|{\bar{E}}_{i}-{\bar{E}}_{j}\right|-\left|{\bar{O}}_{i}-{\bar{O}}_{j}\right|\right)}^{2}$$

To ensure the overall improvement in brightness, the exposure loss was established as Equation (14). The average value of pixels in the pixel block corresponding to the gray-level image of the output-enhanced image should meet certain size requirements, and the reference average value was set to 0.7. ${{\bar{E}}_{m}}^{\prime }$ represents the *m*^th^ pixel value after image channel mean processing and pooling for the enhancement of the final result. The pooling operation may have different parameters. Hence, quotes were added to distinguish it from the spatial consistency loss term. The number of pixels after pooling was set to *M*.


(14) $$L_{\text{exp}}=\frac{1}{M}\overset{M}{\underset{m=1}{\sum }}{\left({{\bar{E}}_{m}}^{\prime }-0.7\right)}^{2}$$

The value of one color channel of the image should not significantly exceed that of the other channels. Hence, the loss of color was set to a constant value represented by $L_{\mathrm{col}}$ represented by Equation (15). This loss should go through all pairings in the three color channels. To better satisfy this condition, the spatial average of the enhanced image is calculated, and a three-channel difference loss term was constructed to satisfy this conclusion. (*c*1,*c*2) traverses all pairwise combinations in the three RGB color channels. ${\bar{E}}^{c1}$ and ${\bar{E}}^{c2}$ represent the enhancement result’s mean value of one RGB channel.


(15) $$L_{\mathrm{col}}=\underset{\forall \left(c1,c2\right)\in c}{\sum }{\left({\bar{E}}^{c1}-{\bar{E}}^{c2}\right)}^{2},c=\left\{\left(R,G\right),\left(G,B\right),\left(B,R\right)\right\}$$

Different from the final enhanced image result, *A* is the network output. In Equation (16), N, which equals to H×W dimensioned by $A_{n}$, represents the shape of the input. d represents the gradient of *A*; for instance, $A_{iy}^{d}$ relates to the longitudinal gradient of *A* in the *i*^th^ iteration. The illumination smooth loss $L_{\mathrm{illu}}$ was established here.


(16) $$L_{\mathrm{illu}}=\frac{1}{N}\overset{n}{\underset{i=0}{\sum }}\underset{d}{\sum }\left(A_{ix}^{d}+A_{iy}^{d}\right)$$

Considering that the brightness change between adjacent pixels is not significant, the gradient term was introduced to the network output to ensure a monotonic relationship between adjacent pixels. No texture was introduced in the network output. Instead, it was introduced from the original image through the relationship. As a common loss term for LLIE, the estimation of the illumination smooth loss term is similar to the calculation of light smoothness loss in Zhang Y. et al. (2019).

## 3 Experiments and evaluation

### 3.1 Experimental setup

The hardware part adopts an 11 GB GTX 1080 Ti. The software is PyTorch framework 1.10.0 v. The spiking encoding convLSTM-augmented LLIE model was constructed using the Python 3.7 library of PyTorch and trained using datasets consisting of unpaired images. The optimization process of the proposed network employed the ADAM optimizer with default parameters and a fixed learning rate of 1 × 10^−4^. The weights *W_col_* and *W_tv_* were set to 0.5 and 20, respectively. These parameters remained constant in all experiments.

The datasets, i.e., LLIE fields, were divided into referenced and unreferenced image datasets. Typical referenced image datasets include LOL, SCIE, and MIT-Adobe FiveK, while unreferenced datasets include VV, NPE, and LIME. The LOL dataset has a considerably different degree of underexposure from the rest, which is suitable for the comparison of the overall performance of LLIE algorithms. The SCIE dataset is a multiexposure image sequence dataset with rich illumination information, which is highly suitable for algorithm debugging. Hence, we selected the LOL and SCIE datasets for the experiments. We retained the original training and test dataset distributions for the LOL dataset. In each image sequence of the SCIE dataset, the first image was chosen as the low-light image to be enhanced, whereas the most suitable one was chosen as the high-light reference image among the third, fourth, and fifth images. We used a user study to evaluate five common unreferenced datasets, namely VV, NPE, LIME, DICM, and MEF. We hypothesized that the key performance of LLIE should lie in the size of the space occupied by its running process, which can influence the integration of related tiny systems. This feature represents the application’s ability to integrate with other functions and algorithms of the testing process and of the model itself.

There are five assessment indices for image objective evaluation, namely peak signal-to-noise ratio (PSNR), structural similarity index measure (SSIM), mean square error (MSE), universal image quality index (UQI) (Wang and Bovik, 2002), and visual information fidelity (VIF) (Sheikh and Bovik, 2006). The calculated evaluation indices are listed in Table 1. In this table, *h,r* corresponds to *H*’s and *R*’s results of the Laplace filter. The *nonzero* (*) function realizes 0 to 1. ∑_gauss_ represents the summation of the results of different Gaussian filter parameters. Gaussian filtering was used for *H* and *R*. The number *n* depicts the Gaussian filtering times. For instance, $\hat{R_{n+1}^{2}}$ indicates that the square calculation was performed first, followed by Gaussian filtering. ${\hat{R_{n+1}}}^{2}$ indicates that Gaussian filtering was performed first, followed by square calculation. $\bar{x}$ represents the uniform filter for *x*. PSNR and MSE are non-negative. Test images with reference images were calculated to get the PSNR value. The larger the PSNR, the less the image noise and the better the image quality, and SSIM reflects structural similarity. It is typically used to measure whether the image backbone of the image recovered by the LLIE has also been restored. The SSIM ranges from 0 to 1; only when two sets of identical image data converge will the SSIM reach 1. The indicator, UQI, reflects the measure of the degree of linear correlation, the closeness of the mean luminance, and the similarity of contrast between the enhanced result and the reference image. VIF combines a natural image statistical model, an image distortion model, and a human vision system model. Compared to the PSNR, SSIM, and other indicators, because the numerator of the VIF index calculation formula is the information fidelity criterion (IFC), VIF has a higher consistency with subjective vision. The higher its value, the better the image quality.

**Table 1:** A calculation of the objective image evaluation index.

Image evaluation index	Mathematical expression	Range	Trend for better
PSNR	$10\log_{10}\frac{\mathrm{MAX}{\left(H\right)}^{2}}{MSE}$	[0,+∞)	To positive infinity
SSIM	$\frac{1}{3}\sum_{\mathrm{channel}}\frac{\left[2\bar{R}\bar{H}+K1\times \mathrm{MAX}{\left(H\right)}^{2}]+[2\hat{R}\hat{H}+K2\times \mathrm{MAX}{\left(H\right)}^{2}\right]}{\left[{\bar{R}}^{2}+{\bar{H}}^{2}+K1\times \mathrm{MAX}{\left(H\right)}^{2}[{\hat{R}}^{2}+{\hat{H}}^{2}+K2\times \mathrm{MAX}{\left(H\right)}^{2}\right]}$	[0,1]	Closer to 1
MSE	$\frac{1}{H\times W}\underset{\left(a,b\right)\in I}{\sum }\left(R_{\mathrm{ab}}-H_{\mathrm{ab}}\right)$	[0,+∞)	Smaller
UQI	$\frac{4\bar{R}\bar{H}\hat{R}\hat{H}}{\left({\bar{R}}^{2}+{\bar{H}}^{2}\right)\left({\hat{R}}^{2}+{\hat{H}}^{2}\right)}$	[−1,1]	Closer to 1
VIF	$\underset{\mathrm{gauss}}{\sum }\underset{\left(a,b\right)\in I}{\sum }\log_{10}\left[1+g^{2}\frac{H^{2}-{\bar{H}}^{2}}{\mathrm{svsq}+2}\right]$	[0, +∞]	Closer to 1

### 3.2 Ablation study

As the proposed method is based on the DCENet structure, the change in the enhancement properties after introducing the spiking+convLSTM structure must be considered. The study demonstrates the influence of each loss term of the loss function on the enhancement results under different loss combinations. In the ablation experiment, different loss combinations were used for retraining. The necessity of each loss item was retested using the proposed DCENet-based method to prevent the negative effects of spiking encoding and convLSTM.

Another ablation experiment should also be considered, which focuses on spiking encoding and convLSTM itself. Thus, three ablation study experiments, whose network is made up of the only light DCENet structure, the structure with the CBAM attention mechanism, or the CNN structure that replaces the convLSTM, have been considered for comprehensively verifying the proposed structure’s necessity. In the two ablation studies, the training parameter did not change. The SCIE dataset was applied for specific calculations.

**Only light DCENet structure**: Without the proposed spiking encoding and convLSTM structure, the enhancement is only realized by DCENet.

**Structure with CBAM attention mechanism**: Based on the only-light DCENet structure, the CBAM attention mechanism is set after the first layer.

**CNN structure that replaces convLSTM**: The enhancement was running using a CNN structure instead of convLSTM. The dimensions of the spiking encoding image sequence were trimmed, and the image sequence was superimposed to form a feature map.

**Figure 4:**
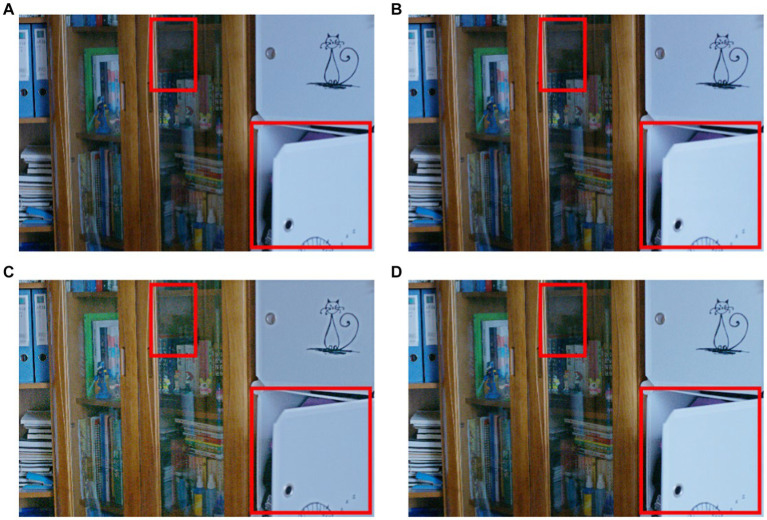
Ablation study by three substructures (obvious areas for specific differences) **(A)** CBAM **(B)** spikingCNN result **(C)** the simple dce structure **(D)** the result obtained by proposed structure.

The ablation study about the importance of spiking encoding and convLSTM is summarized in Table 2. Compared with the basic DCENet structure, spiking combined with the CNN structure revealed that the integration of spiking encoding alone improved the performance. Specifically, the VIF parameter showed significant increments of 9.7 and 4.3% on the LOL and SCIE datasets, respectively. However, when considering other indicators, the objective evaluations of the LOL and SCIE datasets demonstrated a contrary trend. This suggests that the combination of spiking and CNN methods may not be beneficial for enhancing model generalization stability. To improve upon this, the study adopted the classic CBAM attention mechanism as a representative approach for introducing attention mechanisms. Data suggest that incorporating attention mechanisms alone reduced the number of essential evaluation criteria, such as PSNR, SSIM, and UQI. Additionally, the combination of convLSTM and spiking encoding not only elevated the evaluation index on the SCIE dataset but also surpassed the effect of the convolutional network combination. In addition, we identified only minor differences in the subjective effects of the methods under the ablation experiments. These effects are presented in Figure 4.

**Table 2:** The performance comparison of the ablation study for different substructures (red bold for the best, black bold for the second best).

Detection methods	datasets	PSNR	SSIM	MSE	UQI	VIF
SimpleDCE	LOL	17.1200	0.5969	0.0309	**0.7694**	0.8564
	SCIE	**15.4942**	**0.6036**	**0.0337**	**0.8047**	0.5617
Spiking + CNN	LOL	**17.2140**	**0.5999**	**0.0291**	0.7689	**0.9394**
	SCIE	15.1792	0.6007	0.0353	0.7945	**0.5856**
Proposed (Spiking+convLSTM)	LOL	**18.3374**	**0.5974**	**0.0227**	**0.8369**	**1.1019**
	SCIE	**16.7519**	**0.7289**	**0.0221**	**0. 8,413**	0.5358
Proposed + CBAM	LOL	16.9693	0.5968	0.0315	0.7653	0.8539
	SCIE	15.3300	0.6026	0.0347	0.8010	**0.5635**

Ablation experiments assess the impact of different loss function terms on the image enhancement quality. The proposed approach employed four loss function terms. Their pairwise and three-way combinations and the corresponding image evaluation index parameters are listed in Table 3. Of the six paired combination parameters, color constant loss and exposure loss substantially enhanced image quality, followed by spatial consistency loss and exposure loss. Consequently, we infer that exposure loss is the most crucial loss item, followed by color constant loss and spatial consistency loss, which exert the least impact on light smoothness loss.

**Table 3:** Different loss function assemblies of ablation study in the LOL and SCIE datasets (red bold for the best, black bold for the second best).

Lcol	Ltv	Lspa	Lexp	Datasets	PSNR	SSIM	MSE	UQI	VIF
☑	☑			LOL	10.7278	0.3607	0.0940	0.3944	0.2599
				SCIE	9.7752	0.3467	0.1326	0.2811	0.2252
☑		☑		LOL	10.8758	0.3745	0.0908	0.4175	0.2800
				SCIE	10.2279	0.3660	0.1241	0.3243	0.2342
☑			☑	LOL	12.4312	0.4727	0.0670	0.7956	**0.5626**
				SCIE	13.1966	0.4463	0.0602	0.7650	0.7316
		☑	☑	LOL	11.0469	0.4633	0.0844	0.7478	0.4725
				SCIE	11.3607	0.4384	0.0793	0.7144	0.6979
	☑		☑	LOL	8.0262	0.3444	0.1657	0.5044	0.4472
				SCIE	8.6462	0.3565	0.1435	0.4935	**0.8410**
	☑	☑		LOL	10.9274	0.3682	0.0909	0.4029	0.2717
				SCIE	9.8145	0.3491	0.1317	0.2844	0.2273
☑		☑	☑	LOL	**14.2867**	0.5006	0.0470	**0.8284**	0.4368
				SCIE	**14.3752**	0.4610	0.0437	0.7912	**0.8286**
	☑	☑	☑	LOL	8.1473	0.3512	0.1607	0.5078	0.4504
				SCIE	8.6934	0.3591	0.1417	0.4963	0.7412
☑	☑	☑		LOL	10.7671	0.3631	0.0932	0.3984	0.2608
				SCIE	9.8145	0.3491	0.1317	0.2844	0.2273
☑	☑		☑	LOL	13.0447	**0.5859**	**0.0693**	0.8047	0.1875
				SCIE	14.9476	**0.5691**	**0.0335**	**0.8125**	0.7470
☑	☑	☑	☑	LOL	**18.3374**	**0.5974**	**0.0227**	**0.8369**	**1.1019**
				SCIE	**16.7519**	**0.7289**	**0.0221**	**0.8413**	0.5358

**Figure 5:**
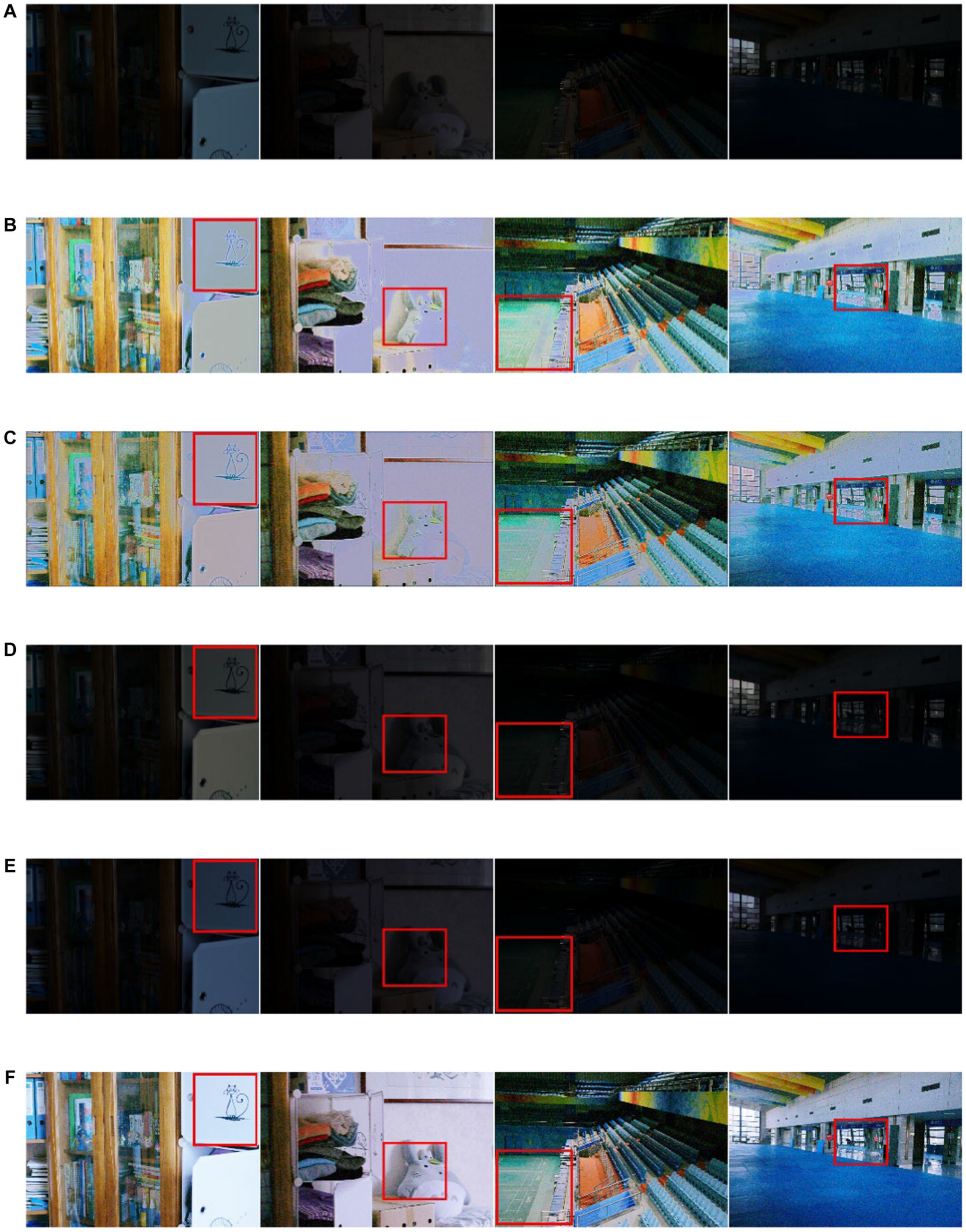

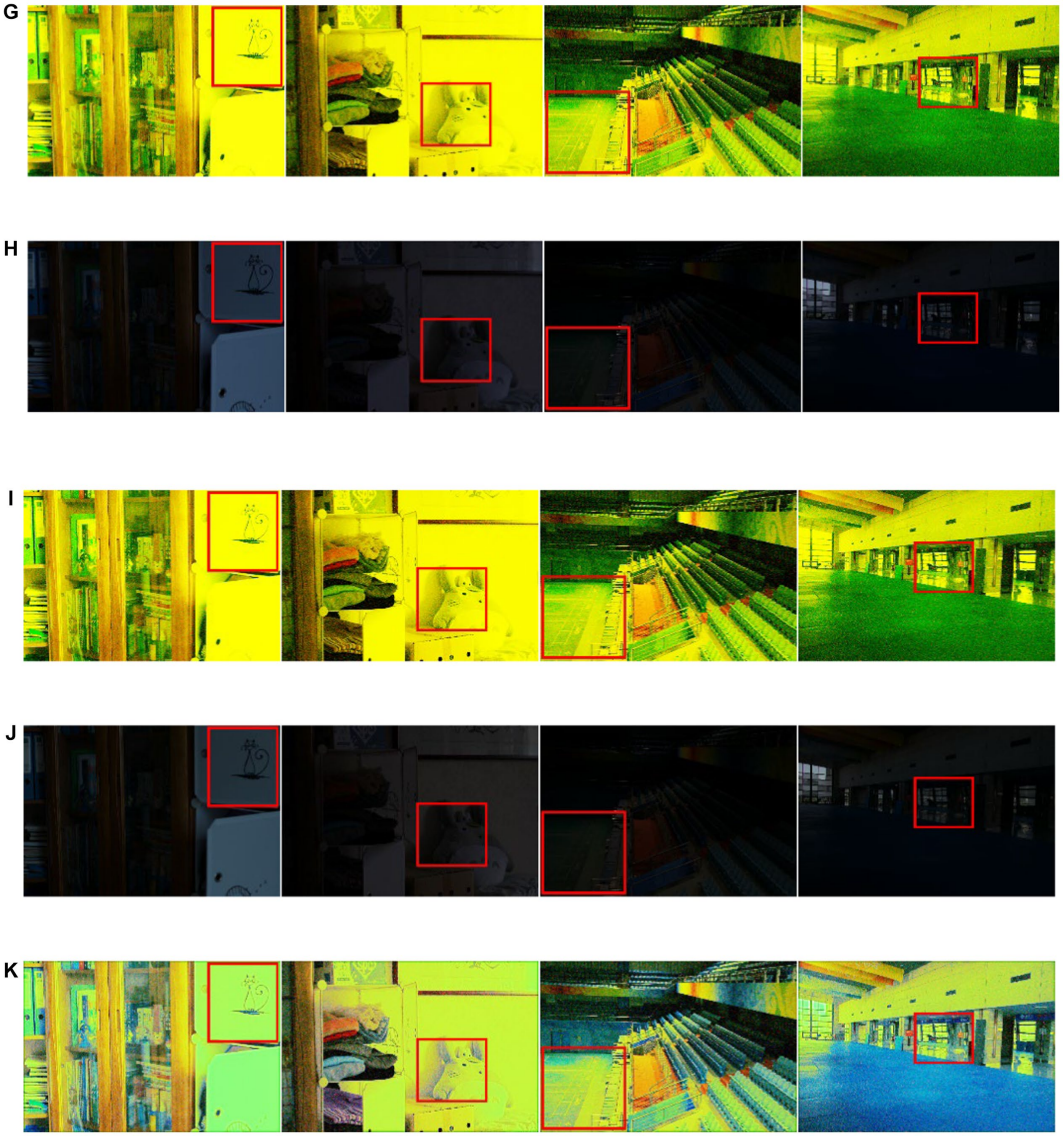
The loss function assembly in LOLdataset **(A)** original low-light image **(B)** color constancy loss and exposure loss **(C)** illumination smooth loss, exposure loss **(D)** color constancy loss, spaital consistency loss, **(E)** spaital consistency loss, exposure loss, **(F)** illumination smooth loss, color constancy loss, **(G)** illumination smooth loss, spaital consistency loss **(H)** illumination loss, exposure loss and color constancy loss **(I)** color constancy loss, exposure loss and spatial consistency loss **(J)** illumination loss, color constancy loss and spatial consistency loss **(K)** illumination loss, exposure loss and spatial consistency loss.

**Figure 6:**
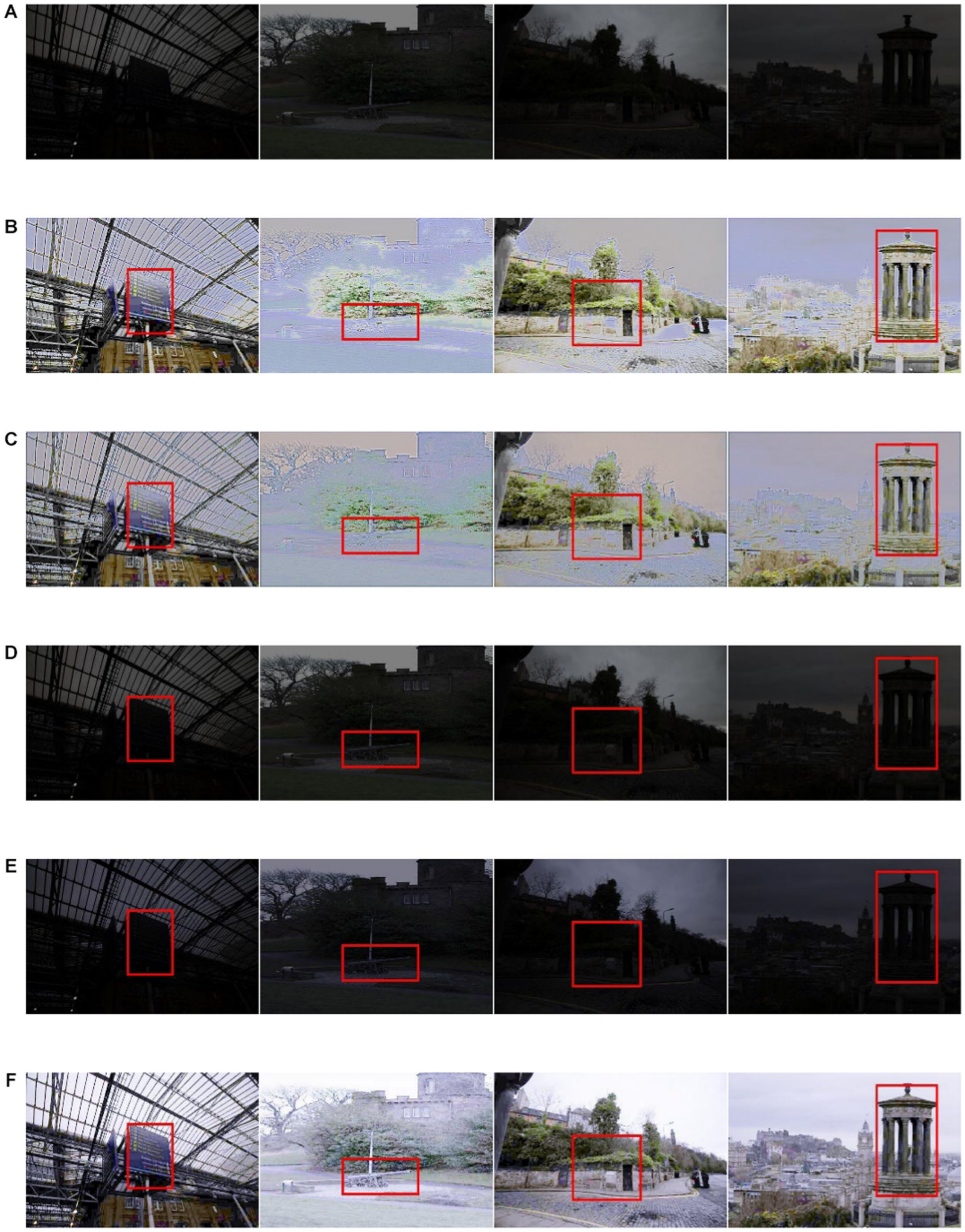

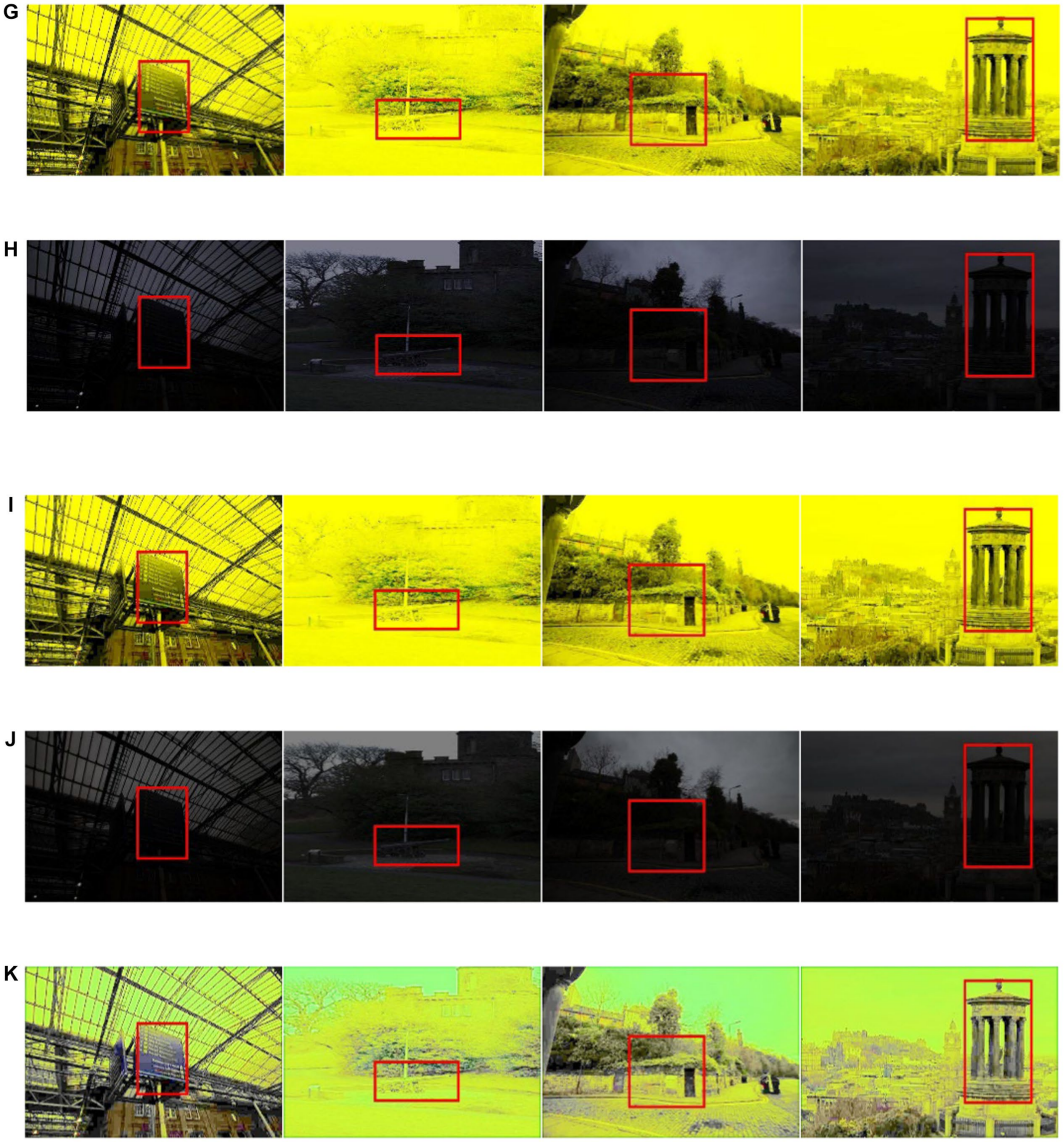
The loss function assembly in SCIE dataset **(A)** original low-light image **(B)** color constancy loss and exposure loss **(C)** illumination smooth loss, exposure loss **(D)** color constancy loss, spaital consistency loss, **(E)** spaital consistency loss, exposure loss, **(F)** illumination smooth loss, color constancy loss, **(G)** illumination smooth loss, spaital consistency loss **(H)** illumination loss, exposure loss and color constancy loss **(I)** color constancy loss, exposure loss and spatial consistency loss **(J)** illumination loss, color constancy loss and spatial consistency loss **(K)** illumination loss, exposure loss and spatial consistency loss.

The study revealed a consistent trend among the four pairs of three-way combination parameters. The method that incorporated exposure loss, color constant loss, and spatial consistency loss outperformed all others in the overall index. However, in terms of UQI, the method combined with exposure loss, color constant loss, and illumination smooth loss performed similarly to the rest. Notably, all four loss functions operated simultaneously. In other words, the index value corresponding to the method proposed in Table 1 is still the best. However, in both the LOL and SCIE datasets, UQI and VIF were marginally inferior to the composite approach of exposure loss, color constant loss, and spatial consistency loss. This highlights the indispensability of using four loss functions. Figures 5, 6 illustrate the influence of each loss function on the image enhancement effect. As observed, exposure loss directly controls image enhancement, while color constant loss mainly controls image distortion after enhancement.

### 3.3 Performance comparison

**Figure 7:**
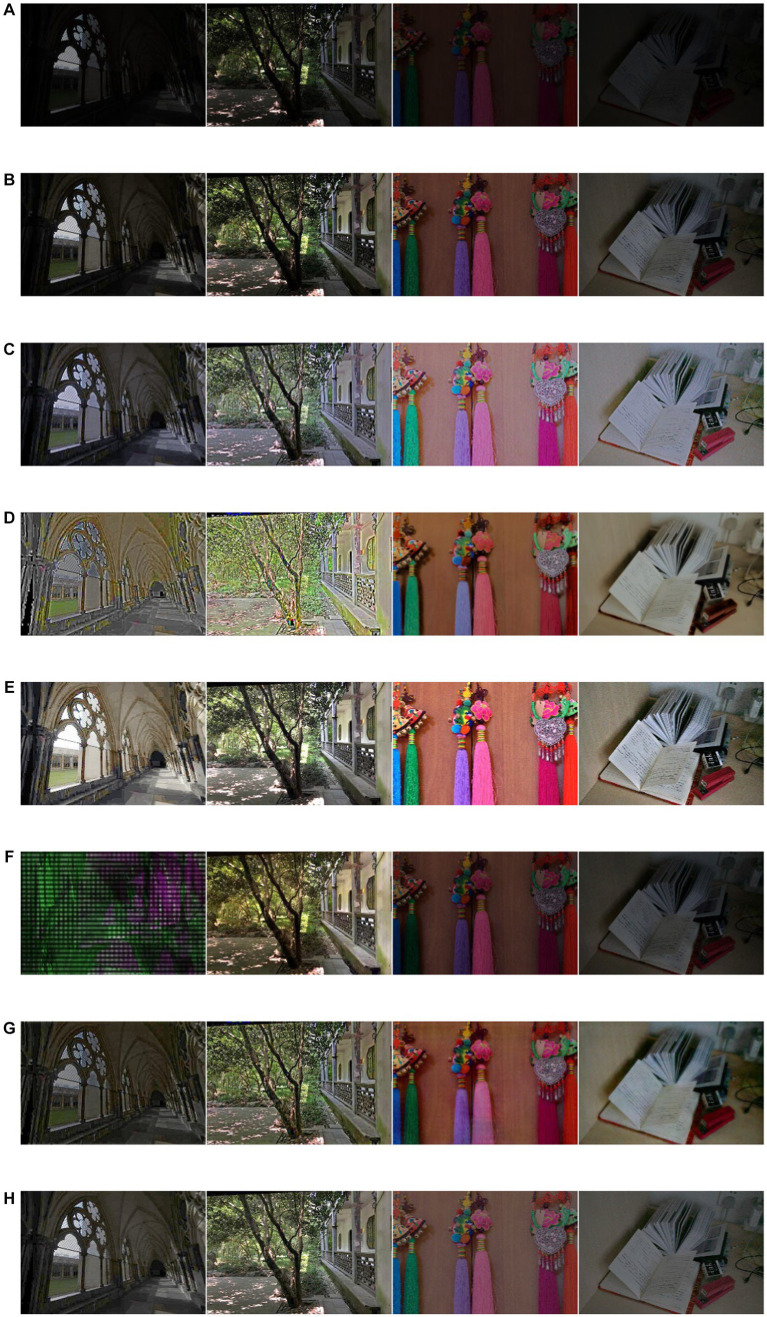

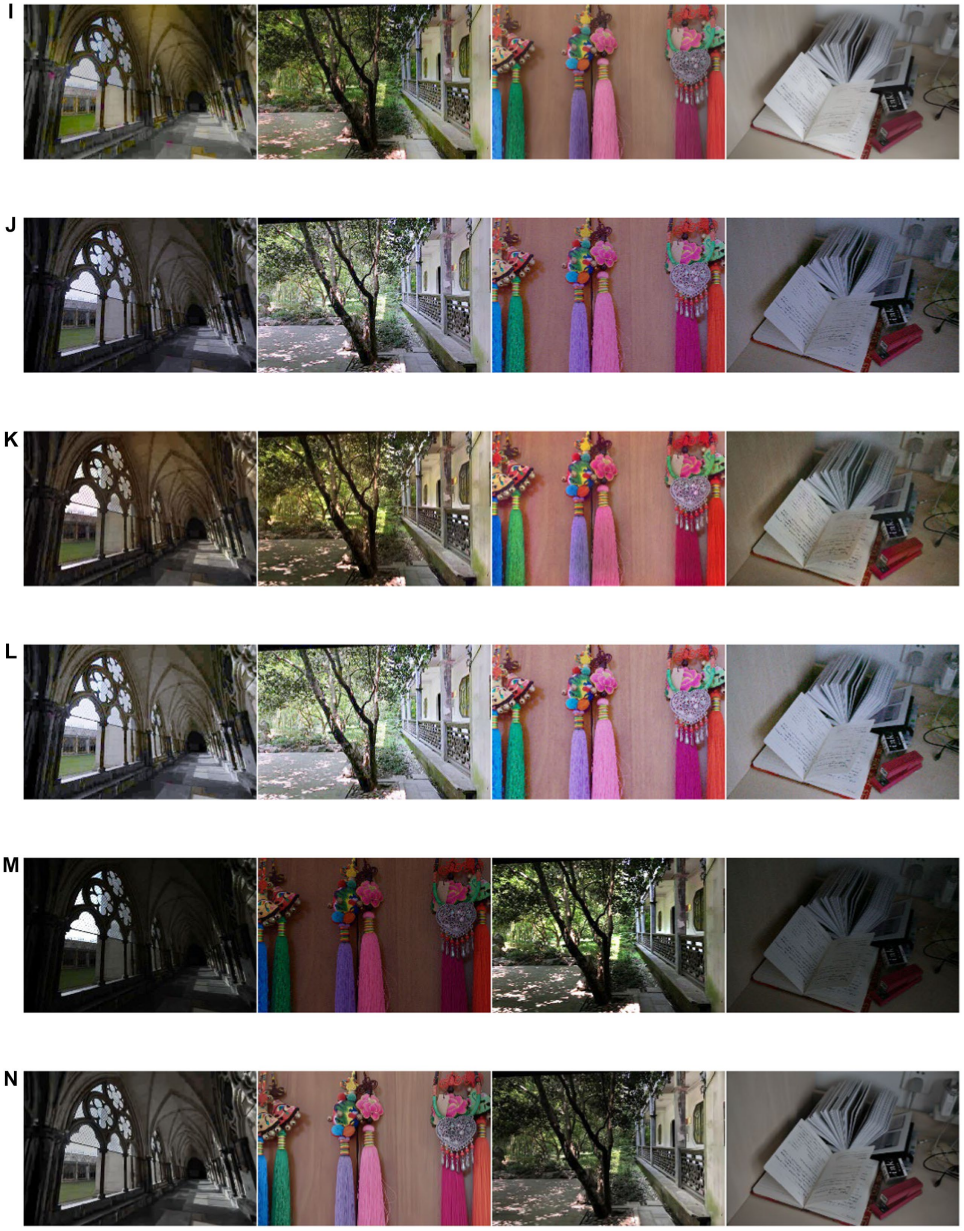
The performance comparsions for different combinations of loss function in SCIE dataset **(A)** original low-light image **(B)** RRDNet **(C)** zerodce **(D)** DRBN, **(E)** EXCNet **(F)** Lightennet, **(G)** DSLR, **(H)** BIMEF **(I)** LLFLOW **(J)** Enlighten Anything **(K)** EnlightenGAN **(L)** Proposed method **(M)** Bread **(N)** BFSA.

After gaining an understanding of the proposed LLIE method, we conclude that the LLFLOW (Wang et al., 2022), BIMEF (Ying et al., 2017), RRDNet (Zhu et al., 2020), zero-DCE (Guo et al., 2020a), DRBN (Yang et al., 2020), EXCNet (Zhang Y. et al., 2019), Lightennet (Zhang et al., 2019), Enlighten Anything (Zhou et al., 2023), EnlightenGAN, DSLR (Ignatov et al., 2017), BREAD (Hu and Guo, 2022), and BFSA (Long et al., 2023) algorithms have strong robustness and potential applications. The proposed method was compared with two referenced datasets in the LLIE field based on five performance indicators. Figure 7 directly demonstrates the enhancement effect. Tables 4, 5 list the performance index values of the proposed method and several of the most popular enhancement methods in the two reference image datasets. The proposed method yielded the best values, with the PSNR, SSIM, MSE, UQI, and VIFP exceeding the second place at 4.4% (0.8%), 3.9% (17.2%), 0% (15%), 0.1% (0.2%), and 4.3% (0.9%), respectively. The numbers inside parentheses represent the increase in the SCIE dataset. In addition, we also measured the parameters related to the actual application characteristics of the resulting algorithm. In the actual application of the image algorithm, the hardware space occupied by the model and the space occupied by the test process warrant attention.

**Table 4:** The performance comparison of the ablation study in the SICE dataset (red bold for the best, black bold for the second best).

Detection methods	PSNR	SSIM	MSE	UQI	VIF
RRDNet	12.4675	0.5469	0.0594	0.5713	0.3487
zero-DCE	16.0794	**0.6618**	0.0365	0.8208	**0.6829**
DRBN	15.8745	0.4667	0.0290	0.8008	0.2575
EXCNet	16.0427	0.6006	0.0334	0.7626	0.3675
LightenNet	11.2150	0.3216	0.0810	0.7228	0.4736
DSLR	15.1002	0.5996	0.0318	0.7839	0.3348
BIMEF	15.7917	0.5904	0.0326	0.7936	0.3678
LLFLOW	15.0300	0.5830	0.0364	0.7918	0.4452
Enlighten anything	14.9228	0.6193	0.0443	**0.8393**	0.4487
Proposed (Spiking+convLSTM)	**16.7519**	**0.7289**	**0.0221**	**0. 8,413**	0.5358
EnlightenGAN	**16.6135**	0.6219	**0.0260**	0.8306	0.5310
BFSA	12.0203	0.4419	0.0721	0.5401	0.5002
Bread	16.0787	0.6209	0.0292	0.7962	**0.6295**

**Table 5:** The performance comparison of the ablation study in the LOL dataset (red bold for the best, black bold for the second best).

Detection methods	PSNR	SSIM	MSE	UQI	VIF
RRDNet	13.1360	0.5598	0.0695	0.5286	0.4951
zero-DCE	17.5592	0.5750	**0.0228**	**0.8355**	**1.0560**
DRBN	17.2850	0.5174	0.0238	0.7222	0.5668
EXCNet	14.8137	0.5539	0.0643	0.7010	0.4894
LightenNet	10.5513	0.1243	0.1183	0.6142	0.1467
DSLR	16.1505	0.6273	0.0389	0.6932	0.5073
BIMEF	17.0586	0.5565	0.0254	0.7792	0.4692
LLFLOW	15.5407	0.5625	0.3552	0.7353	0.5331
Enlighten Anything	16.8056	0.5646	0.0280	0.8678	0.5288
Proposed (Spiking+convLSTM)	**18.3374**	0.5974	**0.0227**	**0.8369**	**1.1019**
EnlightenGAN	17.2322	**0.6945**	0.0287	0.8189	0.7589
BFSA	11.0324	0.4429	0.1031	0.3894	0.3573
Bread	**17.6990**	**0.6530**	0.0273	0.7888	0.8823

Enlighten Anything performs similarly to the EXCNet method. However, it has a good research starting point, which is combined with the large segmentation pretrained model algorithm (Kirillov et al., 2023). Although the LightenNet method meets the characteristics of lightweight, it yields several poor indices. The performance of EXCNet methods is relatively moderate compared to other state-of-the-art methods. The model occupies a relatively large space. In the performance evaluation, the proposed methods, zero-DCE and EnlightenGAN, ranked the highest. The primary role of LLIE methods is to assist with enhancing the realization of other algorithmic functions. Generally, the model and testing process should occupy less space for better integration with other product features. As indicated in Table 6, the space occupied by the proposed algorithm in the test process ranks second, which is only larger than the poorly performing RRDNet, while the space occupied by the model itself reaches 151 KB, which is more than half of the space occupied by the second place.

**Table 6:** Memory occupation of the model and testing process (red bold for the best, black bold for the second best).

Detection methods	Testing memory	Model memory
RRDNet	**1,040,384 (1.04 MB)**	511 KB
zero-DCE	29,149,184 (29.1 MB)	315 KB
DRBN	53,602,358 (53.6 MB)	2.2 MB
EXCNet	22.9 MB (in Tensorflow)	157.2 MB
LightenNet	366.6 MB (in MATLAB)	**108 KB**
DSLR	40,600,532 (40.5 MB)	3.2 MB
BIMEF	125.8 MB (in MATLAB)	/
LLFLOW	239.6 MB	20.9 MB (smallest version)
Enlighten anything	137,830,912 (131.6 MB)	144 MB
Proposed (Spiking+convLSTM)	**22,233,600 (22.23 MB)**	**151 KB**
EnlightenGAN	590,612,992 (590.61 MB)	33,774 KB
Bread	2451.456 MB	6.6 MB
BFSA	3999.744 MB	230.1 MB

### 3.4 User study

Certain LLIE-related datasets have no reference images corresponding to normal light, only images under dark lighting conditions, and therefore it was difficult to use objective evaluation indicators, such as PSNR, to evaluate image quality. To make the performance comparison clearer, more intuitive, and more efficient for these non-reference image datasets, a user study was performed to assess the human perception of the proposed method. The images tested by the user study included various image contents in different environments, including animals, exterior scenes, and buildings. Based on the user feedback data, we constructed a radar map with a maximum of 100 score points for each index, which answered the following five questions:

- Are the details noticeable?
- Are the colors vivid?
- Is the result visually realistic?
- Do the results contain overexposed/underexposed artifacts or over-enhanced/under-enhanced regions?
- Do the results have unnatural texture and noticeable noise?

**Figure 8:**
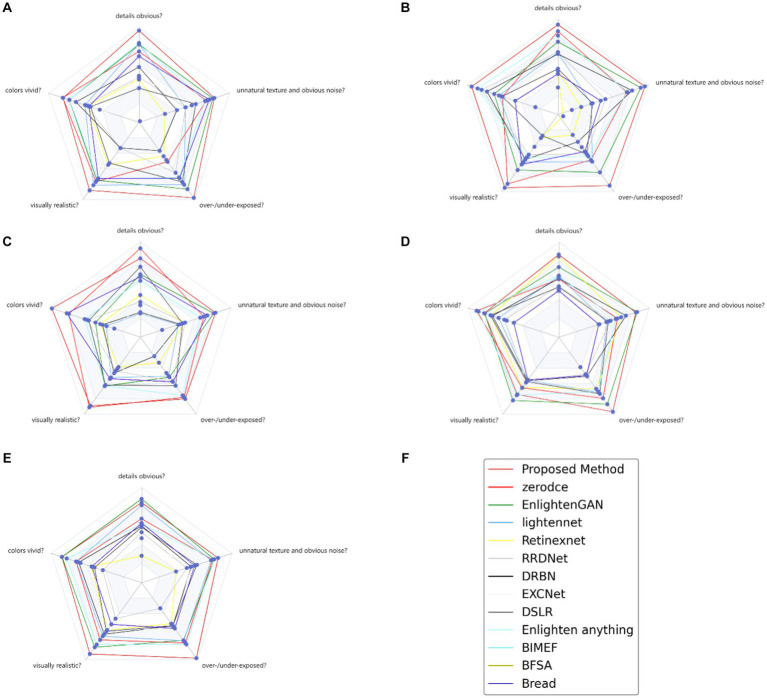
User study for 5 non-reference LLIE datasets **(A)** VV **(B)** NPE **(C)** LIME **(D)** DICM **(E)** MEF **(F)** legend of radar map.

A single radar map can clearly compare the performance of different methods in various aspects of an unreferenced image dataset. The larger the area of the radar map, the better the subjective comprehensive evaluation of the method. Each angular direction, which ranges from 70 to 100 on the radar map, represents the user rating score for a specific problem. The five radar plots in Figure 8 illustrate the distributions of scores evaluated on different questions for different LLIE methods, where the bright red lines in the radar map represent the proposed method. We compared the results of the proposed method for the user study with those of the other LLIE methods using a paired *t*-test (Guo et al., 2020). The results revealed that the effect of EnlightenGAN was the least different from that of the proposed method, except for zero-DCE.

## 4 Discussion

The dark light image enhancement method proposed in this paper has been tested by ablation experiments of different image evaluation indices in different datasets and performance comparison experiments, which have verified its performance superiority. In terms of space proportion, the model in this study is a single model, which does not need to involve a pre-training model or other model framework fusion methods. Enlighten Anything involves the pre-training weight of the SAM model. Compared with EnlightenGAN and related reinforcement learning methods, the new method has relatively low training configuration requirements and difficulty. The limitation of this method is that it is time-consuming at an average of 0.007 s, as determined by the LOL test dataset, which is marginally less than EnlightenGAN. After testing, it was found that the convLSTM structure occupied 0.006 s during testing. However, it still enhances images at 140 fps, which exceeds the real-time demand of 30 fps.

## 5 Conclusion

Originating from the further introduction of spiking coding mechanisms into DL, a novel network exhibits better performance based on DCENet by spiking encoding and convLSTM. Intensity-to-latency conversion, which is a spiking-coding methodology, can be used to gradually acquire the structural characteristics of an image. The multiple subgraphs generated by this method relate to the time step defined by spiking coding, and convLSTM is suitable for solving the image sequence problem and introducing the relationship information between multiple images into the network structure. Furthermore, the simplified DCENet structure without supervision achieved a superior result in terms of improvement. The performance comparison of this method with nine conventional methods in terms of five metrics was validated. The ablation study proved the necessity of the various parts of the structure, such as network and training losses. The proposed method yielded the best values with PSNR, SSIM, MSE, UQI, and VIFP. The proposed model occupies only 151 KB, which will better meet the algorithm integration and practical application requirements on a small chip.

## 6 Scope

The dark light enhancement method used in the study is closely related to the bionic neural networks and learning systems section of the special issue. The relationship between dark light enhancement and neural networks is that neural networks can be applied to tasks with dark light enhancement. Dark light enhancement is an image processing technique designed to improve the visibility of images taken in low-light conditions. By learning a large amount of training data, a neural network can automatically learn and extract the features in the image and perform enhancement processing on the image to improve the quality and visibility of the image.

By using a neural network, a dark light-enhanced model can be built, which is capable of receiving an input image and producing the enhanced image as output. The neural network can automatically learn and fit the mapping relationship between the input image and the output image through the connection and weight adjustment between the multiple layers of neurons. By training and optimizing the neural network, it can enhance the dark light image and have better generalization ability for different input images.

## Data availability statement

The raw data supporting the conclusions of this article will be made available by the authors, without undue reservation.

## Author contributions

XW: Conceptualization, Data curation, Formal analysis, Investigation, Methodology, Software, Validation, Visualization, Writing – original draft, Writing – review & editing. QW: Project administration, Resources, Writing – review & editing. LZ: Conceptualization, Writing – original draft, Writing – review & editing. YQ: Software, Writing – review & editing. FY: Conceptualization, Visualization, Writing – review & editing. JY: Supervision, Validation, Writing – review & editing. QL: Formal analysis, Methodology, Writing – review & editing. RX: Investigation, Validation, Writing – review & editing. ZX: Data curation, Formal analysis, Writing – review & editing. ST: Conceptualization, Formal analysis, Writing – review & editing.
